# Respiratory failure and central nervous system injury caused by mixed gas poisoning after inhaling naphtha vapor: three case reports

**DOI:** 10.3389/fmed.2025.1667174

**Published:** 2025-09-03

**Authors:** Zhaozhao Shan, Jianjian Liu, Ruikai Shang, Hongyu Liu, Qiaoxin Tian, Yuru Liu, Yingying Zheng, Xiangdong Jian, Baotian Kan

**Affiliations:** ^1^Department of Occupational and Environmental Health, School of Public Health, Cheeloo College of Medicine, Shandong University, Jinan, China; ^2^Department of Poisoning and Occupational Diseases, Emergency Medicine, Qilu Hospital of Shandong University, Cheeloo College of Medicine, Shandong University, Jinan, China; ^3^School of Nursing and Rehabilitation, Cheeloo College of Medicine, Shandong University, Jinan, China; ^4^Department of Geriatric Medicine, Department of Nursing, Qilu Hospital, Shandong University, Jinan, China

**Keywords:** naphtha, hydrocarbon, hydrogen sulfide, poisoning, asphyxiating gases

## Abstract

**Objective:**

In March 2025, a rare incident of acute group poisoning due to naphtha vapor inhalation occurred in Shandong, China. This study aimed to analyze the clinical data of patients exposed to mixed asphyxiating gases to enhance awareness of relevant personnel in industrial production and emergency medical staff.

**Methods:**

An on-site investigation and laboratory testing were conducted to examine the poisoning incident. The clinical data of three patients poisoned by asphyxiating gases after inhaling naphtha vapor were retrospectively analyzed.

**Results:**

Patients were primarily exposed to naphtha vapor through the respiratory tract. The main clinical manifestations were respiratory failure and neurological symptoms, such as impaired consciousness. Chest computed tomography and cranial magnetic resonance imaging revealed varying degrees of injury in all three patients, primarily characterized by hypoxic–ischemic brain lesions, pulmonary inflammation, and exudation. Laboratory tests showed arterial blood gas hypoxemia, abnormal white blood cell count, and an increased neutrophil ratio. After mechanical ventilation, glucocorticoid pulse therapy, and neurotrophic treatment, one patient recovered fully within 7 days, one exhibited persistent decorticate symptoms, and one continued to experience respiratory failure requiring mechanical ventilation. All three patients survived.

**Conclusion:**

Inhalation of naphtha vapor led to varying degrees of respiratory failure and neurological impairment in all three patients. Based on on - site sampling analysis and laboratory tests, it was determined that this incident was a poisoning incident caused by inhalation of a mixed gas mainly composed of hydrogen sulfide and alkane gases due to non - compliant operations. Early electrocardiogram monitoring combined with imaging evaluation played a crucial role in guiding clinical management and improving outcomes.

## 1 Introduction

Naphtha is a liquid composed of various alkanes and trace amounts of sulfur-containing compounds. It appears colorless, transparent, or slightly yellow at room temperature and is widely used in chemical production. Naphtha is highly volatile and has a strong aromatic odor. In industrial settings, it can cause poisoning through inhalation via the respiratory tract (1), although cases of absorption through the digestive tract and skin have also been reported (2). The primary causes of death from naphtha exposure include respiratory failure and severe central nervous system injury. Severe poisoning caused by inhaling naphtha vapor is relatively rare. This study reports a case of mixed asphyxiating gas poisoning caused by acute inhalation of naphtha vapor. The incident occurred in a family-operated car washing center, where two workers were cleaning an oil tanker that had previously contained naphtha. The accident caused varying degrees of damage to the respiratory and central nervous systems of 3 patients. The report is as follows:

## 2 Case description

### 2.1 General information about patients and the accident process

**Figure 1:**
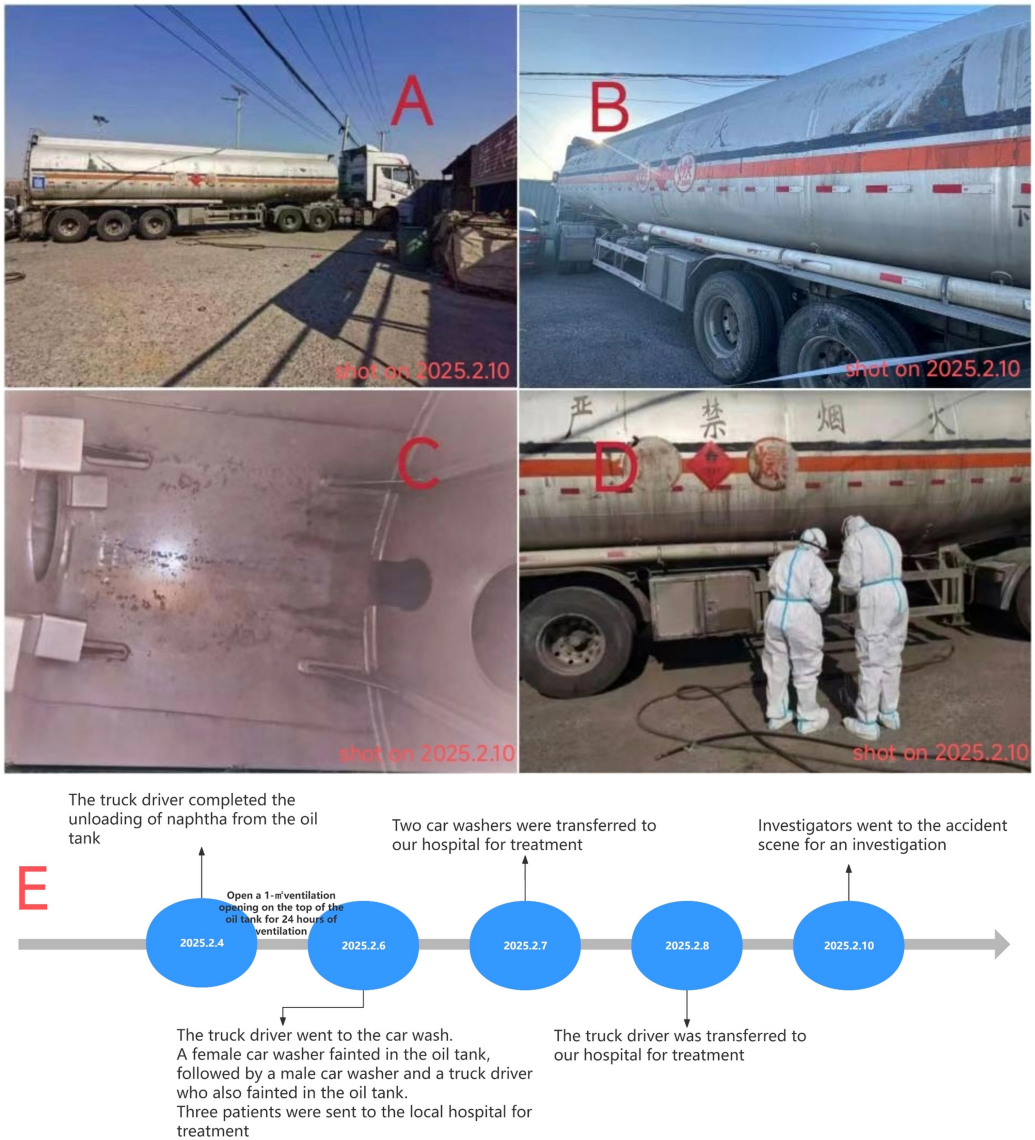
Panels **(A,B)** show the environment at the accident scene; Panel **(C)** displays the interior of the oil tank; Panel **(D)** shows the on-site sampling survey conducted after the accident. These pictures were taken on February 10, 2025. Panel **(E)** show the timeline of the accident.

Three patients were poisoned in this accident. Two of the patients, a man (case 1) and a woman (case 2), were a couple operating a family-run car washing center. The third patient (case 3)was a truck driver transporting hazardous chemicals. On February 4, 2025, the truck driver transported 41 m^3^ of pure naphtha and completed the loading and unloading. After opening a 1 m2 hatch at the top of the oil tank for ventilation for approximately 24 h, on February 6, 2025, the truck driver went to the car washing center run by the couple for interior cleaning of the oil tank. On February 6, 2025, this female car washer, without any protective measures, carried high-pressure water equipment into the oil tanker for cleaning. She lost consciousness immediately upon entering the interior of the oil tank. The male car washer and the truck driver also lost consciousness immediately after entering the tank in an attempt to rescue the female car washer, also without protective measures. Cases 1 and 2 were rescued by a family member, who was wearing a protective mask. The two had been trapped in the oil tank for 30 min and 40 min, respectively. Due to the extreme exhaustion of the family member who performed the rescue, Case 3 was not rescued until 119 emergency responders arrived at the scene an hour later. Case 3 had been in the oil tank for 90 min. The rescuing family member suffered no accidents, and follow-up checks showed no adverse effects. All three patients exhibited signs of unconsciousness and respiratory failure upon being rescued. They were urgently transported to the local hospital for treatment. For further diagnosis and treatment, the three patients were transferred to our department on February 7 and 8, 2025. The accident event line is shown in Figure 1E.

### 2.2 Accident investigation

The incident scene is shown in Figure 1 (These pictures were taken on February 10, 2025). The family car wash center is located outdoors in a relatively simple environment. The couple operating the car wash had not received relevant special training on cleaning hazardous chemical vehicles and lacked awareness of occupational health protection. According to family members, the truck had loaded naphtha on February 4, 2025, and was taken to the car wash on February 6 after the truck driver found residues in the oil tank. On February 10, investigators went to the incident scene and could still detect a pungent odor emitted from the oil tank. Under full protective measures, the investigators sampled the residual liquid in the oil tank and sent it to the molecular testing laboratory for testing. The laboratory results showed that the liquid sample contained alkane components such as ethylcyclohexane, trimethylcyclohexane, and octane. Hydrogen sulfide (H_2_S) gas with a concentration of 8 mg/m^3^ was also detected in the headspace of the sample bottle. Given the 4-day interval between the incident and the on-site investigation, during which the tanker remained in a ventilated state, the findings from the investigation can reasonably be used to determine the nature of the incident.

### 2.3 Changes in the patient’s condition and treatment

**Figure 2:**
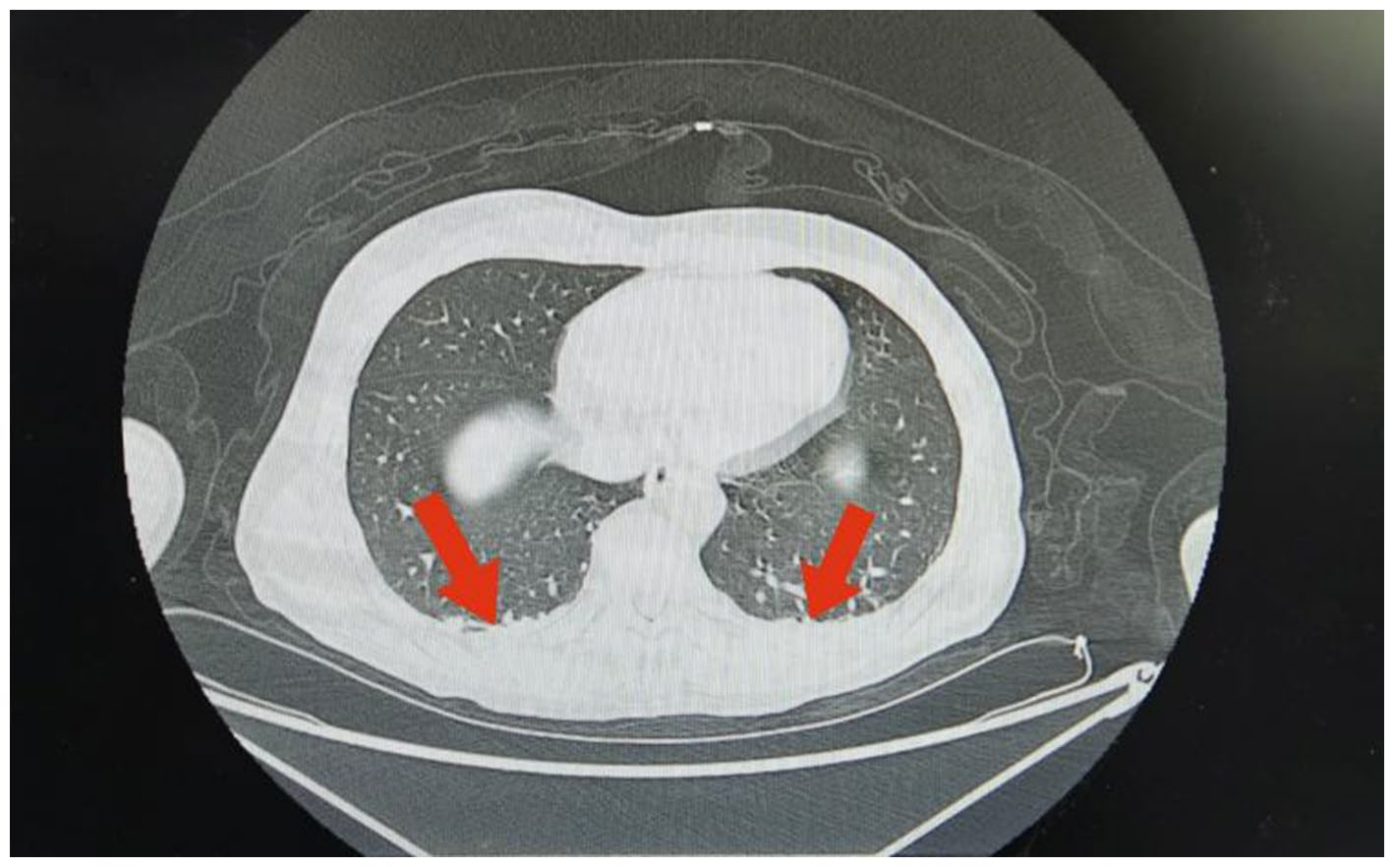
Imaging findings in Case 1: Chest CT shows a small area of inflammation or exudation in the left lung.

**Figure 3:**
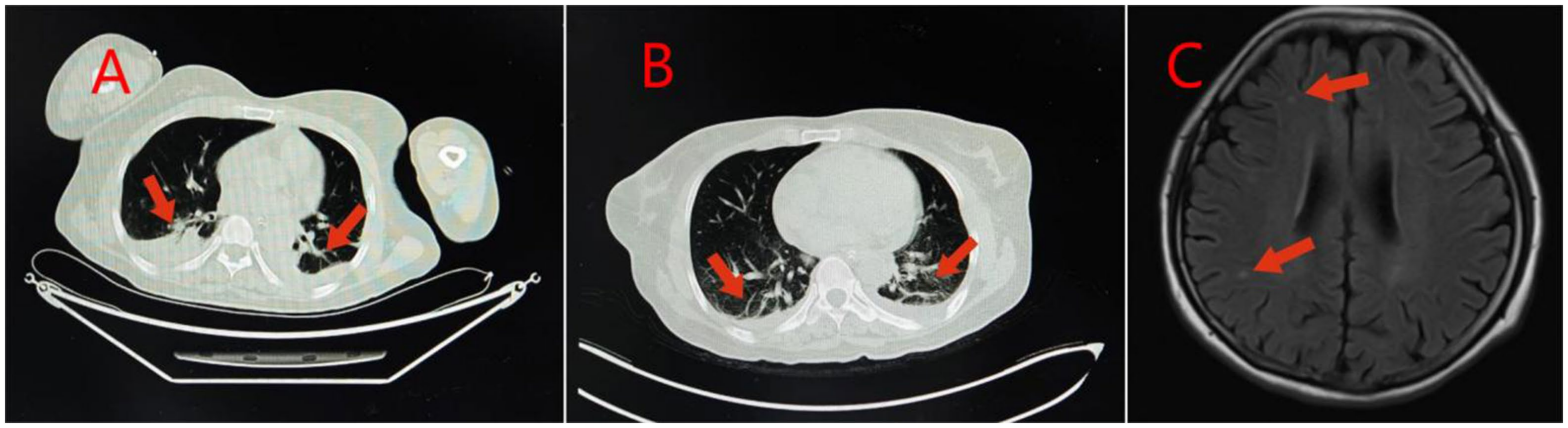
Imaging findings in Case 2: Panel **(A)** Lung CT shows inflammation or hypostatic changes in both lungs, and a small amount of pleural effusion in both pleural cavities; Panel **(B)** Reexamination of lung CT shows inflammation, fibrous foci, and hypostatic changes in both lungs, and a small amount of pleural effusion in both pleural cavities; Panel **(C)** Brain MRI shows a small amount of ischemic degenerative foci in the white matter of both cerebral hemispheres.

**Figure 4:**
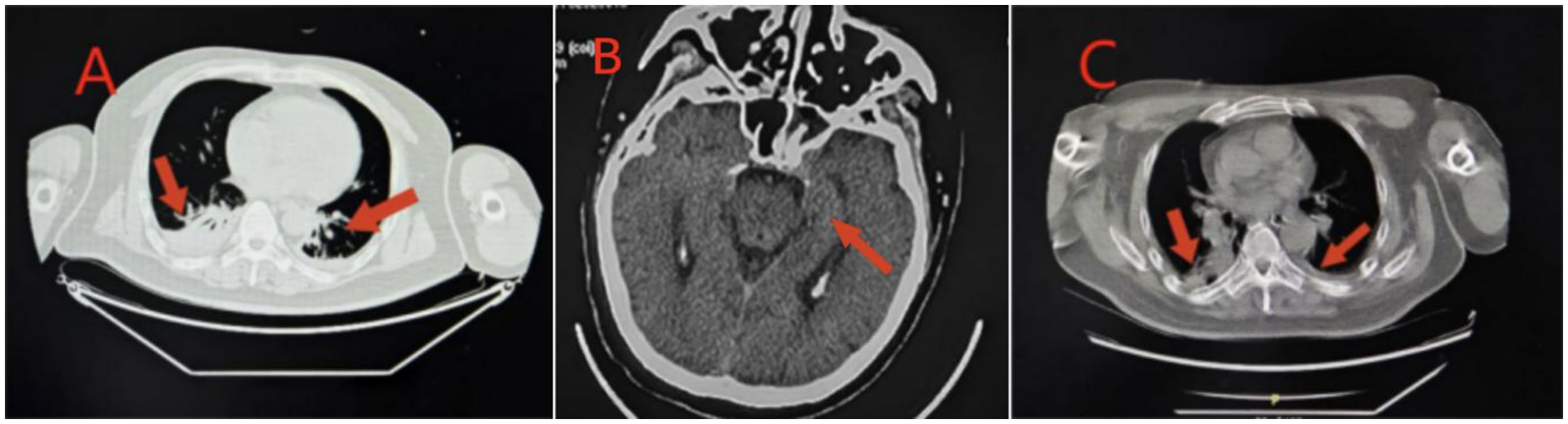
Imaging findings in Case 3: Panel **(A)** Lung CT shows inflammation in the lower lobes of both lungs, which is more prominent on the right side; Panel **(B)** Re-examination of brain CT shows ischemic degenerative foci in the bilateral basal ganglia and corona radiata; Panel **(C)** Lung CT shows inflammation in both lungs, and inflammation in the upper lobe of the right lung.

General patient characteristics, clinical manifestations, and laboratory test results are shown in Tables 1, 2. The results of the CT and MRI examinations are shown in Figures 2–4. The main clinical manifestations were hypoxia and toxic encephalopathy. CT and MRI showed that three patients had varying degrees of pulmonary inflammation, exudation, and hypoxic–ischemic changes in the brain. Laboratory findings showed abnormal white blood cell counts and abnormal neutrophil ratios, while the liver and kidney function indicators were basically normal.

**Table 1:** Main clinical manifestations and examination results of the patients.

Case	Sex	Age	Exposure time	Clinical manifestations at the time of rescue	Chest CT findings	Brain CT findings	MRI findings
1	Male	51	30 min	Unconsciousness, weak breathing	Feb 10: Mild left lung inflammation or exudation	Not performed	Feb 10: No significant brain abnormalities
2	Female	52	40 min	Unconsciousness, cardiac arrest	Feb 19: Bilateral pneumonia or dependent changes; minimal bilateral pleural effusion	Feb 19: No significant brain abnormalities	Not performed
					Mar 1: Bilateral pneumonia, fibrotic foci, dependent changes; minimal bilateral pleural effusion with adjacent atelectasis (decreased compared to Feb 19)	Not performed	Not performed
					Not performed	Not performed	Mar 10: Bilateral cerebral white matter shows minor ischemic degenerative foci
3	Male	52	90 min	Unconsciousness, cyanosis, frothing at the mouth, weak breathing	Feb 18: Bilateral lower lobe pneumonia (right dominant)	Feb 18: No significant brain abnormalities	Not performed
					Mar 6: Bilateral pneumonia, right upper lobe pneumonia (newly developed since Feb 18 CT); bilateral pleural effusion with adjacent atelectasis	Mar 6: Brain CT shows ischemic degenerative foci in bilateral basal ganglia and corona radiata	Not performed

**Table 2:** Laboratory test results of patients at different time points after admission.

Case	Time	WBC (×109/L) 3.5–9.5	NEU (%) 40–75	ALT (IU/L) 9–50	AST (IU/L) 14–40	Cr (μmol/L) 62–115	CK (U/L) 38–174	CK-MB (ng/mL) 0.6–6.3	Detection of unknown poisons in blood
1	2025.2.7	13.37	87.30	47	88	63	3,768	36.2	Not detected
	2025.2.10	9.11	83.10	35	22	78	574	3.5	–
	2025.2.14	10.76	73.40	26	18	66	278	2.1	–
	2025.2.22	7.22	74.60	27	17	77	62	3.9	–
2	2025.2.7	13.50	85.40	50	57	54	1,104	3.7	Not detected
	2025.2.10	12.58	83.50	30	27	41	120	0.9	–
	2025.2.14	16.72	84.80	60	33	45	57	0.9	–
	2025.2.21	14.99	85.50	15	29	42	64	5.7	–
	2025.2.28	9.98	77.30	11	30	37	60	3.8	–
	2025.3.10	6.40	70.70	11	24	41	37	3.4	–
	2025.4.7	7.84	59.40	16	31	35	112	3.2	–
	2025.4.13	6.40	70.10	8	13	42	44	0.9	–
3	2025.2.8	16.49	91.00	77	106	71	363	-	Not detected
	2025.2.10	14.14	82.30	96	39	63	182	1.4	–
	2025.2.14	14.58	80.30	80	40	55	232	5.4	–
	2025.2.21	11.94	74.10	80	48	57	306	3.8	–
	2025.2.28	9.37	66.60	106	56	48	193	2.7	–
	2025.3.7	23.40	85.90	39	25	44	96	2.7	–
	2025.3.16	12.52	73.20	55	35	48	38	1.2	–

WBC, white blood cells; NEU, neutrophils; ALT, alanine transaminase; AST, aspartate aminotransferase; CK, creatine kinase; CK-MB, creatine kinase-MB; Cr, creatinine.

Case 1 was a 51-year-old man with no previous health issues. On admission on February 7, 2025, he presented with weak breathing and loss of consciousness. Physical examination showed a temperature of 36.5 °C, heart rate of 62 beats/min, respiratory rate of 21 breaths/min, blood pressure of 118/75 mmHg, and SpO2 of 99%, revealed a state of drug-induced sedation and tracheal intubation, with no significant abnormalities in other organ systems. Admission diagnosis: Acute poisoning; Asphyxiating gas poisoning. Laboratory and imaging test results are shown in Tables 1, 2 and Figure 2. He was treated with a combination of propofol and dexmedetomidine for sedation; betamethasone to control pulmonary inflammation; danshen polyphenols for cardiovascular protection; and mecobalamin and vitamin B1 for nerve nutrition. Continuous electrocardiogram monitoring was maintained throughout the hospital stay. During the hospitalization, respiratory indicators remained stable, and he was assessed as meeting the criteria for tracheal intubation removal. He transitioned from invasive ventilation with tracheal intubation to low-flow oxygen inhalation and successfully had the tracheal intubation removed on February 9, 2025. The patient’s overall condition was good, and he was discharged on February 14, 2025. A follow-up visit at our hospital on February 22, 2025, showed no significant abnormalities. Telephone follow-up indicated a good prognosis for the patient.

Case 2 was a 52-year-old woman with no previous health issues. She was admitted to the hospital on February 7, 2025, presenting with loss of consciousness and respiratory failure. Physical examination showed a temperature of 36.7 °C, heart rate of 79 beats/min, respiratory rate of 20 breaths/min, blood pressure of 130/81 mmHg, and SpO2 of 98%, revealed a state of drug-induced sedation and intubation, with coarse bilateral breath sounds and a few moist rales. Admission diagnosis: Acute poisoning; Asphyxiating gas poisoning. Laboratory and imaging results are shown in Tables 1, 2 and Figure 3. She was treated with a combination of propofol and dexmedetomidine for sedation; betamethasone and flucloxacillin to control pulmonary inflammation; danshen polyphenols for cardiovascular protection; and mecobalamin and vitamin B1 for nerve nutrition. Continuous electrocardiogram monitoring was maintained throughout her stay. During her hospitalization, her respiratory indicators remained stable, and she gradually weaned off the ventilator. On February 15, 2025, the tracheal intubation was successfully removed, and her spontaneous breathing was normal. However, she was unable to communicate with others and occasionally had convulsions, presenting with a decorticate syndrome. She was discharged on February 30, 2025, and returned to a local hospital for neurological rehabilitation. She was re-examined at our hospital on April 7, 2025, and no significant abnormalities were found in the biochemical tests. Follow-up by phone showed that she was still undergoing neurological rehabilitation treatment.

Case 3 was a 52-year-old man with no previous health issues. He was admitted to the hospital on February 8, 2025, presenting with loss of consciousness and respiratory failure. Physical examination showed a temperature of 36.9 °C, heart rate of 89 beats/min, respiratory rate of 23 breaths/min, blood pressure of 142/89 mmHg, and SpO2 of 99%, revealed a state of drug-induced sedation and intubation, with coarse bilateral breath sounds and audible wet rales. Admission diagnosis: Acute poisoning; Asphyxiating gas poisoning. Laboratory and imaging results are shown in Tables 1, 2 and Figure 4. He was treated with a combination of propofol and dexmedetomidine for sedation; betamethasone and meropenem to control pulmonary inflammation; danshen polyphenols for cardiovascular protection; mecobalamin and vitamin B1 for nerve nutrition; and Xingnaojing injection combined with naloxone to improve cerebral circulation. Continuous electrocardiogram monitoring was maintained throughout the treatment. However, the patient developed refractory inflammation during his hospital stay, which could not be controlled even with the combined use of multiple antibiotics. Additionally, he repeatedly experienced convulsions and restlessness, presenting with decorticate syndrome. Despite having spontaneous breathing, he was unable to be weaned off mechanical ventilation after multiple attempts. On February 20, 2025, a tracheotomy was performed, and the tracheotomy site was connected to an invasive ventilator for assisted ventilation. On March 18, 2025, the family strongly requested to take the patient home for continued treatment at a local hospital. Follow-up by phone indicated that the patient was still receiving treatment at the local hospital.

## 3 Discussion

Naphtha is a petroleum product composed of various alkanes and aromatic hydrocarbons of different molecular weights, along with trace amounts of sulfur. It has a distinct odor and is volatile under normal temperature and pressure. Its components may include cycloalkanes, aromatic hydrocarbons, and H_2_S. Inhaling hydrocarbons such as n-hexane, a constituent of naphtha, can cause arrhythmia, tachycardia, or sudden death. Long-term inhalation of aromatic hydrocarbons in naphtha can damage the brain and the peripheral nervous system (3, 4). The primary cause of death from inhaling naphtha is asphyxiation. Short-term and low-dose exposure can cause cyanosis and hypoxia symptoms, which are often self-limiting (5). Long-term and high-dose inhalation can cause prolonged hypoxia, damage the respiratory and central nervous systems, and ultimately lead to death. Although naphtha poisoning primarily occurs through inhalation via the respiratory tract, cases of poisoning through the digestive tract (6) and intravenous injection (7) have been reported. Naphtha absorbed through the digestive tract can cause acute myocardial infarction and renal failure, manifested as malignant arrhythmia, myocardial ischemia, and kidney injury, likely due to the cardiotoxic effects of alkanes or aromatic hydrocarbon components (8). Pulmonary injury and aspiration pneumonia may also occur, manifested as severe respiratory failure (9, 10). Injecting hydrocarbons into the skin can lead to severe phlebitis and may cause chemical pneumonia or severe hemorrhagic pneumonia (11, 12). Animal studies have demonstrated that exposure to hydrocarbons can induce lipid peroxidation in mice, leading to a decreased level of antioxidant enzymes in the body, accompanied by changes in the morphology of lung tissue, leading to severe lung damage (13). In summary, various poisoning routes of naphtha all show a high degree of harm to the lungs, indicating the pulmonary toxicity of naphtha.

H₂S is a colorless, asphyxiating gas with a strong, pungent odor. It is slightly heavier than air and often accumulates at the bottom of enclosed spaces or areas. It inhibits the cellular respiratory chain (14) and exhibits both neurotoxicity and mucosal corrosive effects, leading to damage in the respiratory and central nervous systems (15). High concentrations of H₂S can directly inhibit the respiratory center of the brainstem, leading to “lightning-like unconsciousness” and respiratory arrest in patients. This further limits the victim’s ability to leave the toxic area and exposes the patient to prolonged poisoning, resulting in damage to multiple organ systems of the body. In severe cases, it can lead to death. Brain injury after H_2_S poisoning usually occurs secondary to severe cardiogenic shock, respiratory depression, and hypoxemia induced by acute pulmonary lesions (16). There is currently no specific antidote for H_2_S poisoning. Clinically, symptomatic support and treatment of complications are mainly adopted, and its prognosis is closely related to the exposure concentration, time, and the speed of emergency intervention. The “lightning-like unconsciousness” during H_2_S poisoning often leads to hasty judgments by rescuers, often resulting in large-scale poisoning incidents (17, 18).

The primary cause of this accident was the presence of high concentrations of H_2_S and other alkane gases in the oil tanker. The patients entered the oil tanker without protective equipment or awareness of occupational safety, leading to their poisoning. All three patients inhaled toxic gases through the respiratory tract, without ingestion through the digestive tract. The relevant toxic substances are metabolized rapidly in the body, and the exposure time at the poisoning site was relatively short. No relevant toxic substances were detected in the blood toxicology tests. In the early stage, all patients showed obvious damage to the respiratory system, accompanied by symptoms of respiratory failure, and were on mechanical ventilation. For the three patients, we promptly administered glucocorticoids to reduce or prevent the occurrence of pulmonary edema and cerebral edema, used neurotrophic drugs such as mecobalamin, and antibiotics to treat the inflammatory response. The duration of toxic gas exposure differed among the three cases. In case 1, the patient had the shortest exposure time and recovered completely without any sequelae after the in-hospital treatment. In Case 2, the patient had moderate exposure time, experienced respiratory recovery during treatment, but developed decorticate syndrome. In case 3, the patient had the longest exposure time, continued to experience refractory respiratory failure, which was difficult to reverse, and also developed decorticate syndrome. These cases highlight that prognosis in gas poisoning is closely related to the exposure time and the speed of emergency intervention.

## 4 Conclusion

In the face of poisoning incidents, medical staff should obtain information on the patient’s poisoning history in a short time, clarify the cause of poisoning, and provide targeted treatment in a timely manner to improve the treatment success rate and reduce the occurrence of sequelae and complications. The frequent occurrence of asphyxiating gas poisoning incidents reflects the existence of loopholes in protection during the labor organization process. Workers’ awareness of protection and protective measures should be enhanced, and strict safety systems and operating procedures should be formulated to prevent similar incidents in the future.

## Data Availability

The original contributions presented in the study are included in the article/supplementary material, further inquiries can be directed to the corresponding authors.

## References

[1] Chen YJ Hsu CC Chen KT. Hydrocarbon pneumonitis following fuel siphonage: a case report and literature review. World J Emerg Med. (2019) 10:69–74. doi: 10.5847/wjem.j.1920-8642.2019.02.001, PMID: 30687441 PMC6340822

[2] Uno M Hongo T Kobayashi S Tamura T. A multidrug therapy for hydrocarbon aspiration with acute respiratory distress syndrome after exposure to oral benzine intake: a case report. Cureus. (2021) 13:e19693. doi: 10.7759/cureus.19693, PMID: 34976473 PMC8681944

[3] Tenenbein M deGroot W Rajani KR. Peripheral neuropathy following intentional inhalation of naphtha fumes. Can Med Assoc J. (1984) 131:1077–9. 6093978 PMC1483777

[4] Tormoehlen LM Tekulve KJ Nañagas KA. Hydrocarbon toxicity: a review. Clin Toxicol (Phila). (2014) 52:479–89. doi: 10.3109/15563650.2014.923904, PMID: 24911841

[5] Wilson FW. Toxicology of petroleum naphtha distillate vapors. J Occup Med. (1976) 18:821. doi: 10.1097/00043764-197612000-00010, PMID: 993875

[6] Lui SZ Niu XY. By naphtha induced pneumonia complicated with empyema: report of one cases. Zhonghua Lao Dong Wei Sheng Zhi Ye Bing Za Zhi. (2012) 30:702–3. doi: 10.3760/cma.j.issn.1001-9391.2012.09.020 23257103

[7] Shusterman EM Williams SR Childers BJ. Soft tissue injection of hydrocarbons: a case report and review of the literature. J Emerg Med. (1999) 17:63–5. doi: 10.1016/s0736-4679(98)00124-3, PMID: 9950390

[8] Roberge RJ Crippen DR Jayadevappa D Kosek TL. Acute myocardial infarction and renal failure following naphtha ingestion. J Emerg Med. (2001) 21:243–7. doi: 10.1016/s0736-4679(01)00378-x, PMID: 11604278

[9] Kamijo Y Soma K Asari Y Ohwada T. Pulse steroid therapy in adult respiratory distress syndrome following petroleum naphtha ingestion. J Toxicol Clin Toxicol. (2000) 38:59–62. doi: 10.1081/clt-100100918, PMID: 10696927

[10] Rajpoot A Sharma P Kumar A Rathore SS. Hydrocarbon pneumonitis with abscess formation following diesel siphoning. BMJ Case Rep. (2022) 15:e249147. doi: 10.1136/bcr-2022-249147, PMID: 35764339 PMC9240896

[11] Neeld EM Limacher MC. Chemical pneumonitis after the intravenous injection of hydrocarbon. Radiology. (1978) 129:36. doi: 10.1148/129.1.36, PMID: 693894

[12] Vaziri ND Smith PJ Wilson A. Toxicity with intravenous injection of naphtha in man. Clin Toxicol. (1980) 16:335–43. doi: 10.3109/15563658008989957, PMID: 7398221

[13] Azeez OM Akhigbe RE Anigbogu CN. Exposure to petroleum hydrocarbon: implications in lung lipid peroxidation and antioxidant defense system in rat. Toxicol Int. (2012) 19:306–9. doi: 10.4103/0971-6580.103678, PMID: 23293471 PMC3532778

[14] Anantharam P Whitley EM Mahama B Kim DS Imerman PM Shao D. Characterizing a mouse model for evaluation of countermeasures against hydrogen sulfide-induced neurotoxicity and neurological sequelae. Ann N Y Acad Sci. (2017) 1400:46–64. doi: 10.1111/nyas.13419, PMID: 28719733 PMC6383676

[15] Santana Maldonado C Weir A Rumbeiha WK. A comprehensive review of treatments for hydrogen sulfide poisoning: past, present, and future. Toxicol Mech Methods. (2023) 33:183–96. doi: 10.1080/15376516.2022.2121192, PMID: 36076319

[16] Sonobe T Haouzi P. Sulfide intoxication-induced circulatory failure is mediated by a depression in cardiac contractility. Cardiovasc Toxicol. (2016) 16:67–78. doi: 10.1007/s12012-015-9309-z, PMID: 25616319 PMC4514577

[17] Zhang ZC Liu JL Jian XD Wang K. An investigation of an accident of occupational acute hydrogen sulfide poisoning. Zhonghua Lao Dong Wei Sheng Zhi Ye Bing Za Zhi. (2017) 35:521–2. doi: 10.3760/cma.j.issn.1001-9391.2017.07.012, PMID: 29081104

[18] Zhao LW Jian TZ Shi LK Li YQ Jian XD Zhang RH. Investigation of an acute hydrogen sulfide mixture gas poisoning in a confined space. Zhonghua Lao Dong Wei Sheng Zhi Ye Bing Za Zhi. (2022) 40:610–2. doi: 10.3760/cma.j.cn121094-20210808-00388, PMID: 36052593

